# A Systematic Review to Assess the Impact of Hyperbaric Oxygen Therapy on Glycaemia in People with Diabetes Mellitus

**DOI:** 10.3390/medicina57101134

**Published:** 2021-10-19

**Authors:** Sudhanshu Baitule, Aaran H. Patel, Narasimha Murthy, Sailesh Sankar, Ioannis Kyrou, Asad Ali, Harpal S. Randeva, Tim Robbins

**Affiliations:** 1 Warwickshire Institute for the Study of Diabetes, Endocrinology & Metabolism, University Hospitals Coventry & Warwickshire NHS Trust, Clifford Bridge Road, Coventry CV2 2DX, UK; narasimha.murthy@uhcw.nhs.uk (N.M.); Sailesh.Sankaranarayanan@uhcw.nhs.uk (S.S.); kyrouj@gmail.com (I.K.); Asad.Ali@uhcw.nhs.uk (A.A.); harpal.randeva@uhcw.nhs.uk (H.S.R.); 2 Warwick Medical School, Faculty of Science, Engineering and Medicine, University of Warwick, Coventry CV4 7AL, UK; 3 Faculty of Health & Life Sciences, Coventry University, Coventry CV1 5FB, UK; 4 Aston Medical School, College of Health and Life Sciences, Aston University, Birmingham B4 7ET, UK; 5 Institute of Digital Healthcare, WMG, University of Warwick, Coventry CV4 7AL, UK

**Keywords:** diabetes, hyperbaric oxygen therapy, glycaemia

## Abstract

*Background and Objectives*: Hyperbaric oxygen is a recognised treatment for a range of medical conditions, including treatment of diabetic foot disease. A number of studies have reported an impact of hyperbaric oxygen treatment on glycaemic control in patients undergoing treatment for diabetic foot disease. There has been no systematic review considering the impact of hyperbaric oxygen on glycaemia in people with diabetes. *Materials and Methods*: A prospectively PROSPERO-registered (PROSPERO registration: CRD42021255528) systematic review of eligible studies published in English in the PUBMED, MEDLINE, and EMBASE databases, based on the following search terms: hyperbaric oxygen therapy, HBO2, hyperbaric oxygenation, glycaemic control, diabetes, diabetes Mellitus, diabetic, HbA1c. Data extraction to pre-determined piloted data collection form, with individual assessment of bias. *Results*: In total, 10 eligible publications were identified after screening. Of these, six articles reported a statistically significant reduction in blood glucose from hyperbaric oxygen treatment, while two articles reported a statistically significant increase in peripheral insulin sensitivity. Two articles also identified a statistically significant reduction in HbA1c following hyperbaric oxygen treatment. *Conclusions*: There is emerging evidence suggesting a reduction in glycaemia following hyperbaric oxygen treatment in patients with diabetes mellitus, but the existing studies are in relatively small cohorts and potentially underpowered. Additional large prospective clinical trials are required to understand the precise impact of hyperbaric oxygen treatment on glycaemia for people with diabetes mellitus.

## 1. Introduction

The use of hyperbaric oxygen in treating decompression sickness in deep-sea divers and people with carbon monoxide poisoning is well-established [1]. Hyperbaric oxygen therapy (HBOT) is also an approved medical treatment for various conditions including necrotizing soft tissue infection, diabetic wounds, osteomyelitis, compartment syndrome, crush and reperfusion injuries, and acute sensorineural hearing loss [1]. HBOT has been postulated to have a positive impact on diabetic foot ulcers, suggesting its incorporation as an adjunct treatment with further scope for research in this area [2,3].

HBOT involves oxygen delivery at a concentration of 100% with a pressure of 2 to 3 atmosphere absolute (ATA) in a hyperbaric chamber. The mechanism of HBOT is to increase tissue oxygen levels resulting in accelerated wound healing, decreased oedema, and killing of anaerobic bacteria [4,5].

In diabetes, meticulous glycaemic control has been shown to reduce the risk of microvascular, macrovascular, and neurological complications [6,7]. There is emerging evidence demonstrating blood glucose level changes in people with diabetes undergoing hyperbaric oxygen treatment [8,9,10,11,12]. However, these studies have involved diverse methodologies, whilst, to date, there has been no systematic review of the impact of HBOT on glycaemia in people with diabetes.

Here, we present the first systematic review considering the effect of HBOT on the glycaemia in people with diabetes. We also explore the proposed mechanisms involved in the potential impact of HBOT on glycaemia in diabetes.

## 2. Materials and Methods

**Figure 1:**
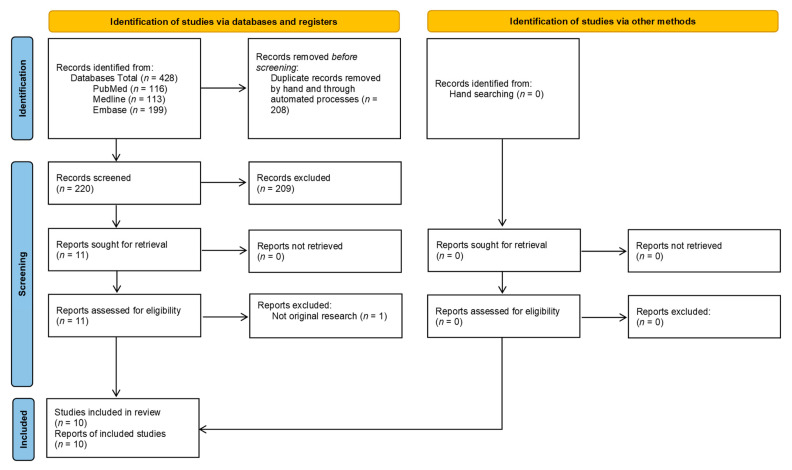
Diagram demonstrating the search strategy used according to the PRISMA protocol [13].

The systematic review was performed according to the PRISMA protocol as shown in Figure 1 [13]. The review was prospectively registered on the NIHR PROSPERO Database (PROSPERO registration ID: CRD42021255528).

### 2.1. Study Selection

The literature search was conducted in the PUBMED, MEDLINE, and EMBASE databases. The search terms used to identify the relevant medical literature were Hyperbaric oxygen therapy/HBO2/hyperbaric oxygenation; Glycaemic control; Diabetes/diabetic/diabetes mellitus; HbA1c. The search strategies used are detailed in the Appendix A (Table A1, Table A2 and Table A3). Only studies involving humans which were published in English language journals were considered eligible, with no restriction to the publication date. Furthermore, filters were applied to set participant’s age as 18 years and above, as this research looked at only the adult population with any type of diabetes (excluding diabetes insipidus) who had undergone HBOT. Any study that focused only on animals, children, or hyperbaric combination therapies was excluded. Any studies focusing on wound care and insulin sensitivity were also excluded. Studies focused on insulin sensitivity but mentioning glycaemia as an outcome in their abstracts were included.

### 2.2. Data Extraction

Data were extracted to a pre-defined, data-extraction proforma which was based on the following variables: year of publication, type of study, location of research and publication, sample size including the baseline characteristics of the population, any biases, single centre or multicentre study, length of follow up comprising of a number of session of interventions, statistical methods used for analysis showing the statistically significant outcome. Miscellaneous variables relevant to this systematic review were also extracted. Data extraction was performed independently by two authors (S.B. and A.P.), with any discrepancies resolved by a third author (T.R.).

### 2.3. Quality Assessment

All studies included in the review were assessed for study quality. Due to the small number of studies and diverse methodologies a single formal tool was not used. Instead, a narrative review was conducted for bias considering sample size, study methodology, any evidence of randomisation, and blinding. All studies were assessed independently for bias by two authors (S.B. and A.P.). Papers were not excluded based on producing a negative outcome or being of low-quality.

### 2.4. Data Synthesis

The diversity among the identified eligible studies, in terms of their study design, sample size and population, did not allow a meta-analysis to be conducted. A qualitative analysis and narrative summary of the studies reporting any change or any factors that pre-dispose to changes in HbA1c were performed. Where possible, these changes have then been grouped under broader categories in a tabulated form.

## 3. Results

The performed systematic search yielded 428 records. Of these, 208 were duplicates and, thus, were removed prior to screening of titles and abstracts. Of the 220 records screened, 11 articles were selected for a detailed review. One of these was excluded after detailed review as it was a letter to editor and not an original research article [10]. In total, 10 studies were eligible for inclusion in this systematic review, which were all available as full text articles. The designs and locations of these studies are summarised in Table 1 and Table 2. The characteristics and findings of the individual eligible studies are summarised in Table 3.

Of the included studies, seven were prospective studies in cohorts of patients with diabetes, two presented retrospective analyses of prospectively collected data, and one study was a randomised, prospective, placebo-controlled trial in patients with type 2 diabetes mellitus. Most of these studies included participants with diabetes mellitus who were receiving HBOT for various indications, including non-healing wounds, diabetic foot ulcers, radio-induced cystitis and neurological deficits such as sudden deafness. 

The majority of the included studies demonstrated a reduction in blood glucose levels following a single session of HBOT in patients with type 2 diabetes mellitus. This effect was consistent across different session lengths and treatment conditions used in the different studies. A prospective cross-over study by Ekanayake & Doolette demonstrated that blood glucose levels in five patients with diabetes mellitus reduced following exposure to both hyperbaric and normobaric conditions, but this decrease only reached significance following exposure to HBOT for at least 45 min [12]. However, this study did not find a significant reduction in blood glucose levels in control subjects without diabetes mellitus in either condition. A significant reduction in blood glucose following a HBOT session in patients with diabetes mellitus was also shown in a prospective study conducted by Trytko & Bennett, which assessed mean blood glucose change across up to 10 consecutive HBOT sessions per participant [9]. This study analysed 226 HBOT sessions across 27 patients, and reported that there was a decrease in blood glucose levels in 80 of the 102 sessions which were in patients with type 2 diabetes mellitus. A prospective cohort study in 23 patients with diabetes mellitus by Al-Waili et al. also demonstrated significant reduction in blood glucose levels as a mean across 15–30 HBOT sessions per participant [19]. Peleg et al. also showed statistically significant decrease in blood glucose levels after a HBOT session in patients with type 2 diabetes mellitus [11], while no significant reduction in blood glucose levels was noted in healthy volunteers without diabetes following HBOT, agreeing with the earlier findings by Ekanayake & Doolette. Moreover, the study by Peleg et al. did not find any significant reduction in blood glucose levels in patients with type 1 diabetes mellitus following exposure to both hyperbaric and normobaric conditions. A retrospective review by Heyboer et al. also found a greater impact of HBOT in patients with type 2 diabetes mellitus as opposed to those with type 1 diabetes mellitus [8]. This retrospective review of prospectively collected data showed that blood glucose levels in patients with diabetes mellitus decreased in 75.4% of 1825 HBOT cycles surveyed. However, on further analysis, a statistically significant greater percentage of treatments of patients with type 2 diabetes mellitus resulted in a decrease in blood glucose levels (77.5%) compared to treatments of patients with type 1 diabetes mellitus (51.5%).

Contrary, the study by Stevens et al. does not support this general finding of a reduction of blood glucose in patients with type 2 diabetes mellitus following HBOT [17]. This retrospective review of prospectively collected data from 190 patients with diabetes mellitus found that in-chamber glucose was higher than pre-HBOT glucose in 54% of sessions. However, there is no evidence in this study of statistical analysis of change in blood glucose levels following HBOT. 

The potential mechanism for a reduction in blood glucose levels caused by HBOT appears to be mediated by increased insulin sensitivity, as opposed to enhanced insulin secretion. The study by Ekanayake & Doolette measured insulin levels in patients with diabetes mellitus during both a single session under hyperbaric and normobaric conditions, and found no change in insulin levels following treatment in either condition [12]. This finding was also seen in the study by Wilkinson, Chapman & Heilbronn; demonstrating that there was no change in fasting insulin levels measured in five patients with type 2 diabetes mellitus even after 30 sessions of HBOT performed over five weeks [18]. This study also demonstrated a statistically significant increase in peripheral insulin sensitivity after both 3 and 30 sessions of HBOT measured using a hyperinsulinaemic clamp in those patients with type 2 diabetes mellitus. A further study by Xu et al. subjected 23 patients with type 2 diabetes mellitus to 30 sessions of either hyperbaric or normobaric conditions and assessed peripheral insulin sensitivity using hyperinsulinaemic-euglycaemic clamps [15]. This study also demonstrated a significant increase in peripheral insulin sensitivity in patients with type 2 diabetes mellitus after 30 HBOT sessions, which was not seen in those exposed to normobaric conditions. However, this study also showed a significant decrease in insulin levels after 30 sessions in both HBOT and normobaric condition groups. This evidence further supports HBOT-induced increased insulin sensitivity as the proposed mechanism for reducing blood glucose levels in patients with type 2 diabetes mellitus.

The reduction in blood glucose levels in patients with type 2 diabetes mellitus attributed to HBOT appears to be longitudinal. The study by Xu et al. demonstrated a significant reduction in fasting plasma glucose after 30 sessions of HBOT [15]. A study by Vera-Cruz et al., which measured fasting plasma glucose and performed an oral glucose tolerance test (OGTT) at baseline and after 20 sessions of HBOT over four weeks, found that whilst fasting plasma glucose did not significantly decrease, there was a significant decrease in glycaemia following an OGTT after 20 sessions of HBOT in patients with type 2 diabetes mellitus [16]. Similarly, Wilkinson, Chapman & Heilbronn also showed no significant reduction in fasting plasma glucose after 30 sessions of HBOT [18]. 

HbA1c was used as an outcome measure in three studies following up participants after multiple sessions of HBOT. The study by Trytko & Bennett found that in 17 patients with type 2 diabetes mellitus who completed 10 sessions of HBOT, there was a small, non-significant reduction in HbA1c [9]. The study by Wilkinson, Chapman & Heilbronn also found no significant change in HbA1c after 30 sessions of HBOT [18]. However, the study by Xu et al. demonstrated a significant reduction in HbA1c after 30 sessions of HBOT, which was not seen in those exposed to normobaric conditions [16]. The study by Irawan et al. corroborates this finding of a reduction in HbA1c, with a significant reduction after 10 HBOT sessions [14].

Some studies suggest that the reduction in blood glucose levels in patients with type 2 diabetes mellitus following HBOT may be independent of the hyperbaric conditions. Peleg et al. found that there was a significant decrease in blood glucose for patients with type 2 diabetes after a session under normobaric control conditions [11]. Ekanayake & Doolette also identified a decrease in blood glucose levels following a session under normobaric conditions. However, this decrease did not reach significance [12]. Both of these studies had relatively small participant numbers and only considered the change in blood glucose after a single session. On the contrary, Xu et al. did not find any change in fasting plasma glucose or HbA1c after 30 sessions under control normobaric conditions [15]. This study by Xu et al. had a larger number of participants and considered the longitudinal impact on glycaemia after multiple sessions. The outcomes measured by Xu et al. could therefore be considered more reliable when considering the clinical utility of HBOT in type 2 diabetes mellitus as a treatment adjunct. However, these discrepancies highlight the need for further controlled trials with larger participant numbers to ascertain the true impact of HBOT on glycaemia in patients with type 2 diabetes mellitus compared to normobaric conditions.

Moreover, four of the studies do have a consideration for the incidence of hypoglycaemic events during or immediately after HBOT. The retrospective review by Heyboer et al. found that none of the patients with diabetes mellitus experienced a hypoglycaemic episode following a HBOT session [8]. However, the retrospective review by Stevens et al. found an incidence of 1.5% for hypoglycaemia during or immediately after HBOT in the 3136 sessions reviewed, but noted that severe or symptomatic hypoglycaemic events were rare [17]. The prospective study by Trytko & Bennett had symptomatic hypoglycaemia occur in 11 out of 237 HBOT treatments in patients with diabetes mellitus; only two of these occurring in patients not requiring insulin treatment [9]. Al-Waili et al. also reported occurrences of symptomatic hypoglycaemic episodes in two of the 41 study participants undergoing HBOT; one of these being an insulin-treated diabetes mellitus patient [19]. Patients with type 1 diabetes mellitus are at an increased risk of hypoglycaemic episodes with HBOT, as found in the study by Stevens et al. and suggested by the results of the Trytko & Bennett study [9,17]. A suggestion is made for a threshold blood glucose level below which the risk of hypoglycaemia during HBOT is increased, with Al-Waili et al. noting this threshold level to be 120 mg/dL, whilst the data from Stevens et al. suggesting that this threshold blood glucose level is 150 mg/dL [17,19].

There are a number of sources of bias to consider when interpreting these studies. The most significant would be the presence of sampling bias. Moreover, most of the studies included for analysis in this review had relatively small sample sizes. Even those with large numbers of HBOT sessions often sourced these from a small number of participants. The small sample sizes used may reduce the reliability of the obtained results and the external validity of the reported findings. These small sample sizes may have also impacted the power of the studies to identify significant changes in glycaemic variables, such as HbA1c, which HBOT may impact in the longer-term.

A number of the studies included patients with both type 1 and type 2 diabetes mellitus that were analysed together in a single diabetes mellitus group. Evidence presented suggests that the effects of HBOT on blood glucose differ between type 1 and type 2 diabetes mellitus when the subgroups are analysed. Therefore, including both patients with type 1 and type 2 diabetes together may impact the demonstrated effects of HBOT. 

The patients included in the majority of these studies received HBOT for treatment of diverse conditions. Common indications for HBOT included non-healing ulcers and diabetic foot ulcers. These indications for HBOT can be associated with poorly controlled diabetes mellitus or long-standing disease, with most studies also having an older age range of participants. These factors could also have an effect on the impact of HBOT on glycaemia.

Another source of bias to consider is that the identified studies have included different HBOT protocols. This included different hyperbaric conditions, different lengths of treatments, and different numbers of treatment sessions per patient. Heyboer et al. have adjusted for this by using each treatment as a unit of analysis as opposed to each participant [8]. Al-Waili et al. and Trytko & Bennet have taken this into account by measuring the mean for each patient as the unit of analysis [9,19]. Trytko & Bennet also found that mean blood glucose reduction following treatment did not significantly alter with treatment number during the course. However, the methodology used by Peleg et al. suggests that the number of treatment sessions is an important factor. Peleg et al. suggest that factors such as anxiety when first introduced to the chamber environment may act as a confounding factor, and so recruited only patients who had already received at least 10 sessions of HBOT to limit this [11]. Ekanayake & Doolette incorporated a similar principle into their methodology for the same reason, only sampling blood glucose and insulin on the third to fifth day of HBOT [12]. Indeed, the findings from the study by Xu et al. also suggest that the number of treatment sessions is influential, with significant changes in insulin sensitivity, HbA1c and fasting plasma glucose only after 30 session of HBOT [15]. Whilst an increase in insulin sensitivity and decreases in fasting plasma glucose and HbA1c were also observed after 10 sessions of HBOT, these changes were not significant.

Finally, two of the prospective studies were cross-over in design [11,12]. Ekanayake & Doolette had all participants exposed to control normobaric conditions on the day of their HBOT session. Peleg et al. had all participants receive their HBOT session before their normobaric session between 1–14 days later. Randomising the sequence of exposures for participants may have helped to ensure that the sequence of exposures is not influencing the results seen.

## 4. Discussion

The impact of HBOT on glycaemia in people with diabetes mellitus is an area of contention as demonstrated by several published studies [8,9,10,11,12]. This review represents the first systematic review conducted on the published research literature to explore the potential impact of HBOT on glycaemic control in people with diabetes. A total of 10 studies were eligible to be included in this systematic review which comprised of seven prospective cross-over and cohort studies; two retrospective reviews of prospectively collected quality data; and one randomised, prospective, placebo-controlled trial.

The majority of the studies demonstrated a reduction in blood glucose levels following HBOT in patients with diabetes, mainly for people with type 2 diabetes mellitus [8,9,15]. The nine original studies reviewed showcased various results involving different methodologies. Variables observed included the use of HBOT and normobaric conditions in people with diabetes mellitus (mainly those with type 2 diabetics mellitus) with assessment of changes in insulin levels, insulin sensitivity, OGTT, and HbA1c (Table 4). Whilst most of the studies support the hypothesis that HBOT reduces blood glucose levels in patients with type 2 diabetes mellitus, there was one study that demonstrated high in-chamber glucose levels contrasting the other study findings [17]. Blood glucose levels, both basal and following an OGTT, were also reduced in people with type 2 diabetes mellitus after several sessions of HBOT [18,19]. Whilst some studies did show a significant reduction in HbA1c following HBOT, the impact seen was not consistent [9,14,15,18]. This highlights the need for large prospective trials to ascertain the precise longer-term effects of HBOT on glycaemia in people with type 2 diabetes mellitus.

The mechanism responsible for the reduction in blood glucose levels caused by HBOT appears to be, at least in part, attributed to increased insulin sensitivity as opposed to enhanced insulin secretion, with two studies demonstrating a significant increase in peripheral insulin sensitivity [15,18]. This finding was particularly noted for people with type 2 diabetes mellitus.

This review has several strengths. These include prospective Prospero registration, independent two author identification, and extraction, a rigorous PRISMA-based approach to reporting, an individualised approach to quality assessment of each paper, no restriction to publication date.

However, there are also certain limitations to consider regarding this systematic review. Indeed, this research only considers the literature published in the English language, thus excluding relevant studies that may have been published in other languages. Accordingly, only 10 studies published in the English language were eligible for inclusion, whilst the small sample sizes included in these studies may reduce the reliability and generalisability of the relevant findings. Finally, due to the small sample sizes and diverse methodology involved in the eligible studies, a meta-analysis was not possible, and rather, an individualised approach to conducting this systematic review was considered.

## 5. Conclusions

This systematic review suggests that HBOT can impact glycaemia for people with diabetes. Indeed, this systematic review brings together articles demonstrating the impact of HBOT in lowering blood glucose and improving insulin sensitivity in people with type 2 diabetes mellitus. Despite these findings, there remains uncertainty as to the clinical significance of these HBOT-induced effects on glycaemic control. There is, therefore, a need for further research to consider the longer-term clinical impact of HBOT on glycaemia for people with type 2 diabetes mellitus, which could be considered as a potential adjunctive therapy to potentially improve glycaemic control in selected cases.

## Figures and Tables

**Table 1:** Summary of study designs included in this systematic review.

Study Design	Number of Studies	Percentage of Studies (%)
Prospective cohort	7	70
Randomised placebo-controlled trial	1	10
Retrospective analysis	2	20

**Table 2:** Summary of locations (countries) of the studies included in this systematic review.

Study Location	Number of Studies
United States	3
Australia	3
China	1
Indonesia	1
Israel	1
Portugal	1

**Table 3:** Summary of the characteristics and findings of the ten eligible original research studies included in this review. DM = Diabetes Mellitus HTN = Hypertension, HBOT = Hyperbaric oxygen therapy, HbA1c = Glycated haemoglobin, IQR = Interquartile range, OGTT = oral glucose tolerance test, T1DM = type 1 diabetes mellitus, T2DM = type 2 diabetes mellitus.

	Data Collection	Study Design	Study Location	Sample Size	Sub-Population	Length of HBOT	Controls	Statistically Significant Outcomes	Other Notable Outcomes
Heyboer et al.2019 [8]	Single Centre	Retrospective analysis	United States	77 patients	Patients with diabetes mellitus undergoing HBOT for various indications	Median 19 sessions (IQR = 31)	None	Statistically significant greater percentage of treatments of patients with T2DM resulted in a decrease in blood glucose levels (77.5%) vs. T1DM (*p* < 0.001)	Blood glucose decreased in 75.4% of treatments in this group with a median decrease of 25 mg/dL (IQR = 54 mg/dL)
Irawan et al. 2018 [14]	Single Centre	Prospective cohort study	Indonesia	15 patients	Patients with diabetes mellitus and diabetic foot ulcers	10 sessions	No HBOT	Significant decrease in HbA1c after 10 session from 10.98 ± 2.37 % to 9.70 ± 2.46 % (*p* = 0.006)	None
Xu et al. 2017 [15]	Single Centre	Randomised, prospective, placebo controlled	China	23 patients	Patients with T2DM suffering from intracerebral haemorrhage	30 sessions	Normobaric oxygen therapy	A significant increase in insulin sensitivity during the HBOT sessions after 30 sessions (*p* < 0.05). Significant decreases in insulin, fasting glucose (11.3 ± 1.5 vs. 9.6 ± 1.1 mmol/L), and HbA1c (9.2 ± 1.6 vs. 7.8 ± 1.3%) in the HBOT group after 30 sessions (*p* < 0.05)	No change in insulin sensitivity, fasting plasma glucose of HbA1c in normobaric conditions.
Vera-Cruz et al. 2015 [16]	Single Centre	Prospective cohort study	Portugal	16 patients	Patients with T2DM and indications for HBOT	20 sessions	Patients without T2DM	Glycaemia measured following OGTT significantly decreased from 280.25 ± 22.29 mg/dL to 185.78 ± 11.70 mg/dL after 20 sessions of HBOT in patients with T2DM	HBOT decreased fasting plasma glucose levels to 119.1 ± 4.80 mg/dL in patients with T2DM, however without reaching statistical significance (*p* = 0.089)
Stevens et al. 2015 [17]	Single Centre	Retrospective analysis	United States	190 patients	Patients with diabetes mellitus receiving HBOT for various indications	1 session	None	None relevant	In-chamber glucose was higher than pre-HBOT glucose in 1708 of the 3136 HBOT sessions (54%)
Peleg et al. 2013 [11]	Single Centre	Prospective cohort crossover study	Israel	13 patients	Patients with insulin- and non-insulin-dependent diabetes mellitus with HBOT indicated for non-healing wound	1 session	Room air conditions at sea level pressure13 patients with traumatic brain injury or stroke treatedfor neurological deficit13 healthy volunteers	The non-insulin dependent diabetes mellitus patients had a significant decrease in their blood glucose levels during both sessions; from 9.2 ± 3.0 mmol/L to 7.3 ± 3.0 mmol/L during HBOT and from 9.9 ± 2.9 to 7.8 ± 3.4 mmol/L (*p* = 0.004) during the control normobaric session	The insulin-dependent patients had no change in blood glucose either during HBOT (13.0 ± 4.0 mmol/L before to 13.2 ± 5.7 mmol/L after, *p* = 0.88) or during the control session (13.15 ± 2.7 before to 13.2 ± 4.7 mmol/L after, *p* = 0.96)
Wilkinson et al. 2012 [18]	Single Centre	Prospective cohort study	Australia	5 patients	Obese patients with T2DM and indications for HBOT	30 sessions	Non-obese individuals without T2DM	Peripheral insulin sensitivity was significantly increased by HBOT at 3 and 30 visits in patients with T2DM. (*p* = 0.008).HbA1c wassignificantly reduced only in subjects without diabetes (*p*< 0.05	No significant change in HbA1c after 30 visits in patients with T2DM. No change in fasting plasma glucose and insulin after 30 visits
Al-Waili et al. 2006 [19]	Single Centre	Prospective cohort study	United States	23 patients	Patients with diabetes mellitus and indications for HBOT	15–30 sessions	None	HBOT caused a significant dropin mean blood glucose approximately to the same extent in patients with diabetes mellitus alone or in patients with both diabetes mellitus and hypertension	Significant drop in blood glucose in 12 patients without HTN, and diabetes mellitus.
Trytko & Bennet. 2003 [9]	Single Centre	Prospective cohort study	Australia	27 patients	Patients over 18 years old with diabetes mellitus and indications for HBOT	Up to 10 consecutive sessions	None	Mean reduction in blood glucose for each individual following HBOT of 2.04 (*p* < 0.0001)	T2DM were 102 of the recorded sessions and 80 of these had a reduction in blood glucose. Mean blood glucose reduction following HBOT did not significantly alter with treatment number during the course. In 17/23 patients who completed 10 sessions, there was a small and non-significant reduction in the mean HbA1c by 0.22% (*p* = 0.06)
Ekanayake & Doolette. 2001 [12]	Single Centre	Prospective cohort crossover study	Australia	5 patients	Patients with diabetes mellitus of >6 years duration and indications for HBOT	1 session	Normobaric air conditions 5 patients without diabetes mellitus	Decline in glucose levels in both HBOT and normobaric conditions in patients with diabetes mellitus. Decline only reaches significance between time points after 45 min in HBOT	No change in serum insulin levels under any condition

**Table 4:** Summary of the eligible studies reviewed with their outcomes for this systematic review.

Year	Study Reference	Impact on Fasting Blood Glucose	Change in Insulin Levels	Peripheral Insulin Sensitivity	Impact on OGTT 2-h Glucose Level	HbA1c Change
2019	Heyboer et al. [8]	Decrease *	NA	NA	NA	NA
2018	Irawan et al. [14]	NA	NA	NA	NA	Decrease *
2017	Xu et al. [15]	Decrease *	Decrease *	Increased *	NA	Decrease *
2015	Vera-Cruz et al. [16]	Decrease	NA	NA	Decrease *	NA
2015	Stevens et al. [17]	Increase ^†^	NA	NA	NA	NA
2013	Peleg et al. [11]	Decrease *	NA	NA	NA	NA
2012	Wilkinson et al. [18]	No change	No change	Increased *	NA	No change
2006	Al-Waili et al. [19]	Decrease *	NA	NA	NA	NA
2003	Trytko & Bennet. [9]	Decrease *	NA	NA	NA	Decrease
2001	Ekanayake & Doolette. [12]	Decrease *	No change	NA	NA	NA

* Indicates statistical significance. ^†^ There is no evidence in this study of statistical analysis of change in blood glucose levels following HBOT. OGTT = Oral Glucose Tolerance Test, HbA1c = Glycated haemoglobin. Green background colour indicates a positive impact, red background colour indicates a negative impact and an orange background colour indicates no impact.

## Appendix A

**Table A1:** Demonstrating the search strategy used on the PubMed database.

Search Number	Query	Results
17	#15 AND #16	116
16	#3 AND #6	931
15	#7 OR #8 OR #9 OR #10 OR #11 OR #12 OR #13 OR #14	624,093
14	“hypoglycemia”[MeSH Terms]	28,951
13	hypoglycaemia[Title/Abstract] OR hypoglycemia[Title/Abstract] OR hypoglycaemic[Title/Abstract] OR hypoglycemic[Title/Abstract]	59,161
12	“hyperglycemia”[MeSH Terms]	37,247
11	hyperglycaemia[Title/Abstract] OR hyperglycemia[Title/Abstract] OR hyperglycaemic[Title/Abstract] OR hyperglycemic[Title/Abstract]	65,507
10	“glycemic control”[MeSH Terms]	270
9	glycemic[Title/Abstract] OR glycaemic[Title/Abstract]	50,821
8	“blood glucose”[MeSH Terms]	168,266
7	“blood sugar”[Title/Abstract] OR glucose[Title/Abstract]	502,819
6	#4 OR #5	16,145
5	“Hyperbaric Oxygenation”[Mesh]	12,056
4	“hyperbaric oxygen*”[Title/Abstract] OR HBO2[Title/Abstract] OR HBO[Title/Abstract]	12,164
3	#1 OR #2	729,572
2	“Diabetes Mellitus”[Mesh]	440,904
1	diabetes[Title/Abstract] OR diabetic*[Title/Abstract]	669,965

**Table A2:** Demonstrating the search strategy used on the Medline database.

#	Query	Results
S17	S15 AND S16	113
S16	S3 AND S6	919
S15	S7 OR S8 OR S9 OR S10 OR S11 OR S12 OR S13 OR S14	597,728
S14	(MH “Hypoglycemia+”)	28,914
S13	TI (hypoglycaemia OR hypoglycemia OR hypoglycaemic OR hypoglycemic) OR AB (hypoglycaemia OR hypoglycemia OR hypoglycaemic OR hypoglycemic)	58,437
S12	(MH “Hyperglycemia+”)	37,174
S11	TI (hyperglycaemia OR hyperglycemia OR hyperglycaemic OR hyperglycemic) OR AB (hyperglycaemia OR hyperglycemia OR hyperglycaemic OR hyperglycemic)	63,950
S10	(MH “Glycemic Control”)	257
S9	TI (glycemic OR glycaemic) OR AB (glycemic OR glycaemic)	50,265
S8	(MH “Blood Glucose”)	167,965
S7	TI (“blood sugar” OR glucose) OR AB (“blood sugar” OR glucose)	473,206
S6	S4 OR S5	15,885
S5	(MH “Hyperbaric Oxygenation”)	12,048
S4	TI (“hyperbaric oxygen*” OR HBO2 OR HBO) OR AB (“hyperbaric oxygen*” OR HBO2 OR HBO)	11,723
S3	S1 OR S2	727,311
S2	(MH “Diabetes Mellitus+”)	439,985
S1	TI (diabetes OR diabetic*) OR AB (diabetes OR diabetic*)	663,799

**Table A3:** Demonstrating the search strategy used on the Embase database.

#	Query	Results
17	15 and 16	199
16	3 and 6	1303
15	7 or 8 or 9 or 10 or 11 or 12 or 13 or 14	864,384
14	exp hypoglycemia/	82,561
13	(hypoglycaemia or hypoglycemia or hypoglycaemic or hypoglycemic).ti. or (hypoglycaemia or hypoglycemia or hypoglycaemic or hypoglycemic).ab.	86,203
12	exp hyperglycemia/	101,038
11	(hyperglycaemia or hyperglycemia or hyperglycaemic or hyperglycemic).ti. or (hyperglycaemia or hyperglycemia or hyperglycaemic or hyperglycemic).ab.	94,423
10	exp glycemic control/	53,870
9	(glycemic or glycaemic).ti. or (glycemic or glycaemic).ab.	81,559
8	exp glucose blood level/	260,424
7	((blood adj1 sugar) or glucose).ti. or ((blood adj1 sugar) or glucose).ab.	658,030
6	4 or 5	14,749
5	exp hyperbaric oxygen/ or exp hyperbaric oxygen therapy/	3230
4	((hyperbaric adj1 oxygen*) or HBO2 or HBO).ti. or ((hyperbaric adj1 oxygen*) or HBO2 or HBO).ab.	13,775
3	1 or 2	1,195,748
2	exp diabetes mellitus/	1,014,391
1	(diabetes or diabetic*).ti. or (diabetes or diabetic*).ab.	995,738
